# Cognitive status and foot self care practice in overweight diabetics, engaged in different levels of physical activity

**DOI:** 10.1186/2251-6581-13-31

**Published:** 2014-02-04

**Authors:** Farah Madarshahian, Mohsen Hassanabadi, Mohsen Koshniat Nikoo

**Affiliations:** 1 Nursing Department, Birjand University of Medical Sciences, Ghafary Avenue, Birjand 9717853577, Iran; 2 Community and Public Health Department, Birjand University of Medical Sciences, Ghafary Avenue, Birjand 9717853577, Iran; 3 Endocrinology and Metabolism Research Center, Endocrinology and Metabolism Clinical Sciences Institute, Tehran University of Medical Sciences, North Kargar Street, Tehran 1411413137, Iran

**Keywords:** Diabetes mellitus type 2, Exercise, Cognition Disorders, Body mass index, Self care

## Abstract

**Background:**

Type 2 diabetes along with chronic hyperglycemia may result in cognitive impairment. This can negatively affect the patient’s adherence to diabetes treatment. The purpose of this study was to compare the cognitive status and foot self care practice in overweight type 2 diabetic patients who exercised regularly and those who did not.

**Methods:**

The comparative study was conducted on 160 consecutive patients from an outpatient diabetes clinic. They were divided into two groups: The active group comprised of 80 patients engaged in regular exercise for at least 15–30 minutes, three times per week during the past 6 months. The control group included 80 patients who had not exercised regularly for the past 12 months, matched for sex, age, education, diabetes duration, hemoglobin A1C and body mass index (BMI: 25–29.9Kg/m^2^). Data on the patients’ demographic information, foot care practice and physical activity habits were gathered using a questionnaire. The Mini Mental Status examination (MMSE) was applied to assess cognitive status.

**Results:**

MMSE score was significantly higher in the active group. A significant negative correlation was noted between MMSE scores and BMI in the control group (r = −0.2, *P =* 0.03). A significant difference was noted in the four domains of foot self care practice between the active (4.77 ± 0.77) and control (4.45 ± 0.83) groups (*P <* 0.01).

**Conclusions:**

Regular physical activity can help promote cognitive status and foot self care practice in overweight patients with type 2 diabetes.

## Introduction

The prevalence of type 2 diabetes is increasing worldwide. The condition along with chronic hyperglycemia commonly found in the sufferers may result in cognitive impairment mainly in executive functions, memory, attention and psychomotor activity and disability in long term [1,2]. This can negatively affect the patient’s adherence to diabetes treatment [3].

Type 2 diabetes is also associated with obesity, hypertension and dyslipidemia; all which can damage the brain [4,5]. Longitudinal studies indicate that high total fat mass and central adiposity increase the risk of developing cognitive impairment in the elderly with type 2 diabetes [6]. In addition a recent Chinese cross-sectional study has showed that; pre-diabetes or diabetes, obesity, particularly central obesity are associated with cognitive impairment in individuals of different age groups [7].

The etiology of cognitive impairment in type 2 diabetics is uncertain. Some studies suggested adipose tissue secretes factors such as adipokines that contribute to systemic and vascular inflammation, development of atherosclerosis and diabetes complications [8]. Obesity-induced cognitive impairment may be an adverse outcome of vascular defects, impaired insulin metabolism or defect in glucose transport mechanisms in the brain [9]. Hyperglycemia and high glycosylated hemoglobin (A1C) levels [1] can therefore cause cerebrovascular disease and cognitive impairment [10].

Regular physical activity can be beneficial in patients with type 2 diabetes. Evidence from longitudinal studies and trials suggest that exercising [11,12] along with cognitive training promote cognitive function in the elderly [13]. Aerobic exercise improves cognitive function in older adults with glucose intolerance, [14] particularly those aged 75 and over [15].

Growing body of evidence supports the health benefits of regular exercising both in healthy subjects and those with diabetes. Regular engagement in physical activity reduces the risk of developing cerebral and cardiovascular disease through improving cerebral blood flow [16], reducing the levels of hemoglobin A1C, an index of blood glucose control, improving lipid profile, lowering weight and body fat mass [17-19].

In addition, physical exercise promotes cognitive function in dementia persons [20]. In a case–control study, getting engaged in moderate physical activity reduces the likelihood of experiencing mild cognitive impairment at any age [21]. Therefore it could be concluded that exercising can help prevent or improve cognitive function [22,23]. This comes while several studies have found contradictory results [24].

Self care practice is a key point in diabetes management with foot self care being one of the most important parts of this practice. Exercising postpones the development of peripheral neuropathy in these patients. This comes while impaired sensation, commonly reported as a complication in diabetic patients, may increase the risk of developing infected foot ulcers during exercise and thus reduce exercising capacity [25].

Diabetes educators need to ensure patients being engaged in appropriate exercise and be aware of proper foot care practice [26]. Thus continuous education about foot self care practice is necessary in diabetics [27]; cognitive dysfunction and obesity may interfere with foot self care in these patients. Few studies however have been conducted in this regard [7]. Therefore the purpose of this study was to compare the cognitive status and foot self care practice between two groups of overweight patients with type 2 diabetes who exercised regularly and those who did not.

## Methods

### Study participants and design

In this comparative study, all consecutive overweight patients with type 2 diabetes who attended the diabetes clinic of the Endocrinology and Metabolism Research Institute affiliated to Tehran medical University in 2010 were recruited. The diagnosis of diabetes was made for all the patients by one of the endocrinologists of the diabetes clinic [28,29]. Being overweight was defined as having Body Mass Index (BMI) levels between 25 and 29.9 Kg/m^2^.

“Diabetes duration” was defined as the time between the diagnosis of diabetes mellitus and when the MMSE examination was performed. Patients who were diagnosed with diabetes in less than one year ago were excluded [29]. Illiterate patients and those with less than 8 years of schooling, current smokers, those with severe vision and hearing problems, patients with a positive history of stroke, Alzheimer disease, psychiatric problems [28], any endocrine disorders that could interfere with cognition, and severe cardiovascular disease [30] that could interfere with MMSE testing or were known risk factors for cognitive impairment were excluded.

The active group comprised of eighty overweight patients with type 2 diabetes, who exercised regularly for at least 15–30 minutes three times a week during the past six months. The control group included eighty overweight diabetic patients who did not exercise regularly in the past 12 months (no exercise or exercising for less than three times per week, less than 45 minutes per week, or about 45 minutes but not regularly and weekly). Two trained clinical nurses unaware of the objectives of the study were responsible for selecting the active group from among overweight diabetic patients who exercised regularly at least during the past 6 month. One patient with type 2 diabetes meeting control group qualifications were recruited from the same diabetes clinic for each active group participant. The two groups were matched according to sex, age, educational class, diabetes duration, body mass index and hemoglobin A1C, through group matching. It must be mentioned that all of the participants took oral hypoglycemic agents. The participants were interviewed by the nurses after they signed the informed consents.

### Measurements and study variables

The two trained nurses recorded the patients’ demographic information, foot self care practice and physical activity status using a questionnaire. Cognitive status was assessed using the MMSE questionnaire. Schooling was quantified according to education years. The participants’ history of physical and mental (depression, dementia and Alzheimer disease) disease was performed through Diagnostic and Statistical Manual of Mental Disorders Text Revision criteria (DSM-IV-TR). A psychologist confirmed the results.

Exercise is a subcategory of physical activity. According to the Centers for Disease Control and Prevention [31], regular physical activity was defined exercising was considered as: being engaged in any physical activity (walking, bicycling, swimming, hiking, weight training, aerobics or other exercises) for at least 15–30 minutes [22], 3 times per week during the past 6 months [31-33].

The participants of this study who report that they did the same physical exercise for at least prior 6 months were classified as active group and those who did not regular exercise for at least past 12 month, as control group [34]. In this study physical exercise was calculated in minutes (minutes of exercise at a time by the times per week that participants did exercise).

The nurses measured the patients’ body weight using a scale (Seca 700, Vogel, & Halke, Germany) with a 220 kg maximum capacity, sensitive to 50 gr, and height without shoes accurate to 1 cm, using a wall-mounted stadiometer (Seca 220, Vogel, & Halke, Germany).

Mini Mental Status Examination (MMSE) Questionnaire is introduced as a standard measure of cognitive function for both research and clinical purposes. MMSE consists of 19 questions and tests five areas of cognitive function: orientation, registration, attention, calculation, recall, and language. The normal score in this test is “between” 24 and30, lower values indicate cognitive impairment (0–17: severe and 18–23: mild to moderate cognitive impairment). The validity and reliability of MMSE has been approved in previous studies [28].

Venous blood samples were obtained after overnight fasting and were analyzed on the same day in the central laboratory of the research center.

Foot self-care practices were assessed using self-reports addressing recommended foot self-care among diabetic patients based on four questions. The questions include: (1) How many times in the last 7 days did you wash your feet? (2) How many times in the last 7 days did you dry between your toes after washing? (3) How many times in the last 7 days did you check your feet? and (4) How many times in the last 7 days did you inspect the inside parts of your shoes? In this regard“0 days” meant (foot self-care was never performed during the past week), and “7 days” meant (foot self-care done every day during the past week) [35]. The validity and reliability of the scale has been approved in previous studies [36].

### Statistical analysis

Independent T test was used to compare age, duration of diabetes, foot care, BMI and MMSE scores between the two groups. Chi square test was used to compare the literacy status and sex between two groups. Pearson correlation test was used for MMSE scores and minutes of exercise per week in active group. Logistic regression was performed to predict age, duration of diabetes, BMI, foot care and sex as independent variables on MMSE scores as dependent variable in two groups. MMSE scores were considered as dichotomous variable: below 24 (moderate and severe cognitive impairment) and 24 and higher (normal cognitive status). Data were analyzed using the statistical package for social science (SPSS software version 16, Inc., Chicago, IL, USA). For analysis, P ≤ 0.05 was considered as statistically significant.

### Ethical aspects

This research was approved by the ethic committee of the Endocrinology and Metabolism Research Institute of Tehran University of Medical Sciences (N:00151). Each participant provided written consent and filled in questionnaires anonymously. Participant data were associated with numbers rather than participant names.

## Results

One hundred and sixty overweight diabetic men and women (age range 55–80, Mean age: 63.38 ± 6.60) participated in this research. Table 1 shows the demographic characteristics of the patients and their MMSE scores.

**Table 1:** Characteristics, Demographic and MMSE score of patients with type 2 diabetes and overweight; with and without regular exercise

**Variable**	**Active group**	**Control group**	**df**	**x2/ T**	**P**
	**N = 80**	**N = 80**			
Age* (y)	63.66 ± 6.37	63.1 ± 6.85	158	−0.53	0.59
**Sex (n%)**					
Female	43(53.75)	42(52.5)	1	0.025	0.87
Male	37(46.25)	38(47.5)			
Diabetes duration* (y)	9.56 ± 2.92	9.65 ± 2.93	158	0.18	0.85
**Education (n%)**					
High school	55(8.75)	56(70)	1	0.029	0.86
Higher	25 (31.25)	24(30)			
BMI*	27.49 ± 0.88	27.32 ± 0.87	158	−1.24	0.21
**HbA1**_C_* (mmol/lit)	7.13 ± 0.16	7.17 ± 0.23	158	1.50	0.13
MMSE Score*					
MMSE total	28.2 ± 2.18	25.23 ± 1.75	158	−8.08	0.001
MMSE in female	28.2 ± 2.4	25.19 ± 1.77	83	−6.94	0.001
MMSE in male	28.2 ± 1.98	25.28 ± 1.76	73	−5.58	0.001
Foot care practice*	4.77 ± 0.77	4.45 ± 0.83	158	−2.54	0.01
(day/week**)**					

*Data for these variable are mean ± SD.

Note. P ≤ 0.05 is considered significant, Mini Mental State Examination (MMSE), Active group: patients with regular exercise. Control group: patients without regular exercise, Body Mass Index: 25–29.9 Kg/m^2^.

The mean time spent on regular exercise was 173.18 ± 70.79 minutes per week in the active group (160.69 ± 61.04 in women and 187 ± 79.02 in men). Mean MMSE score in the participants who did 90–150 minutes of exercise was 26.70 ± 2.13, 151–300 minutes of exercise was 29.28 ± 1.2 and 301–450 minutes of exercise was 30. There was a significant positive correlation between MMSE scores and minutes spent on exercising each per week (r = 0.55, *P =* 0.01).

There was no correlation between MMSE scores and BMI in the active group (r = 0.037, *P =* 0.74). A significant negative correlation was noted between MMSE scores and BMI in the control group (r = −0.2, *P =* 0.03). Table 2 shows mean MMSE scores and foot care practice based on BMI in two groups.

**Table 2:** The mean of MMSE scores and foot care based on BMI in two groups

**Participants**	**Active group (n = 80)**			**Control Group (n = 80)**		
	**BMI**	**MMSE**	**Foot care**	**BMI**	**MMSE**	**Foot care**
BMI Kg/m^2^	n (%)	(mean ± SD)		n (%)	(mean ± SD)	
25-26.99	11(13.75)	27.1 ± 2.2	4.6 ± 0.8	33(41.25)	23.3 ± 1.6	4.4 ± 0.8
27-28.99	62(77.5)	28.3 ± 2.1	4.7 ± 0.7	43(53.75)	25.3 ± 1.7	4.4 ± 0.8
29-29.99	7(8.75)	26.8 ± 2.4	4.7 ± 0.7	3(5)	23.0 ± 2.0	4.3 ± 0.3

Note. Body mass index (BMI): 25–29.9 Kg/m^2^, Active group: patients with regular exercise. Control group: patients without regular exercise, Mini Mental State Examination (MMSE); 0–17: severe cognitive impairment (there is no patient with severe cognitive impairment in this study) , 18–23: mild to moderate impairment, 24–30: normal.

The mean number of times dedicated to foot care practice during the past week was 4.77 ± 0.77 and 4.45 ± 0.83 in the active and control group respectively (P = 0.01). The mean number of times dedicated to foot care during the past week in women was 4.86 ± 0.82 and 4.30 ± 0.89 respectively in active and control group (P = 0.004) and in men was 4. 68 ± 0.72 and 4.61 ± 0.73 respectively in active and control group (P = 0.70). Table 3 shows the number of foot self-care practices in the two groups.

**Table 3:** Foot self-care practices in two groups

**Foot self-care practice**	**Active group**				**A.G**	**Control group**				**C.G**		
					**mean ± SD**					**mean ± SD**		
(Day/week) n (%)	0	1-3	4-6	7		0	1-3	4-6	7		t	P
Washing of feet	0(0)	2(2.5)	68(85)	10(12.5)	5.22 ± 1.04	1(1.2)	7(8.7)	70(87.5)	2(2.5)	4.77 ±1.20	−2.53	0.01
Drying in between toes	4(5)	20(24.9)	52(65.1)	4(5)	4.08 ± 1.59	5(6.2)	18(22.5)	57(71.2)	0(0)	3.81 ± 1.27	−1.20	0.23
Checking of feet	1(1.2)	12(15)	3(66.3)	14(17.5)	4.85 ±1.56	2(2.5)	7(8.7)	65(81.3)	6(7.5)	4.78 ±1.33	−2.71	0.78
Inspecting inside of shoes	0(0)	10(12.5)	56(70)	14(17.5)	4.95 ± 1.33	6(7.5)	4(5)	65 (81.2)	5(6.2)	4.43 ± 1.58	−2.21	0.02

Table 4 shows the relationship between BMI, age, duration of diabetes and sex to MMSE.

**Table 4:** Odds Ratio of some variables to Mini Mental State Examination (MMSE) of patients with type 2 diabetes and overweight; with and without regular exercise

**Variable**	**Active group**	**Control group**	**OR**	**95% CI**	**P**
		**N = 80**	**N = 80**		
**Sex n (%)**					
Male	37(46.25)	38(47.5)	Reference		
Female	43(53.75)	42(52.5)	0.63	0.15-2.55	0.52
**Age (y) n (%)**					
55-65	53(66.2)	54(67.5)	Reference		
66-80	27(33.8)	26(32.5)	7.62	1.74-33.43	0.007
BMI*	27.49 ± 0.88	27.32 ± 0.87	2.25	1.04-4.85	0.03
Duration of diabetes* (y)	9.56 ± 2.92	9.65 ± 2.93	1.27	1.01-1.59	0.03
**Foot care n (%)**					
0- 4	42(52.5)	27(33.8)	1.99	0.46- 8.49	0. 35
4.1- 7	38(47.2)	53(66.2)	Reference		

*Data for these variable are mean ± SD.

Note. Body mass index (BMI): 25–29.9 Kg/m2, Active group: patients with regular exercise. Control group: patients without regular exercise.

## Discussion

The purpose of this study was to compare the cognitive status and foot self care practice between two groups of overweight diabetics engaged in different intensities of exercise.

The present study showed higher MMSE scores in the active group, revealing that exercising may improve cognitive function. Mounting evidence shows that exercising has positive effects on cognitive function [22,23]. Its mechanism of action however is not clearly known. It is possible that exercising can improve cognitive function through promoting cerebral vascular function and brain perfusion through improving cerebral blood flow [16]. Moreover the cardiovascular fitness associated with exercising can improve the plasticity of the aging brain, and thus reduce both biological and cognitive senescence [37]. In line with these findings, a previous study of brain function in diabetic and healthy individuals has shown that physical activity with mild to moderate intensity improves the brain function [38]. In addition, the results of randomized controlled trial on twenty-eight adults (57–83 years old) found a cognition-enhancing effect for six months of aerobic exercise in older glucose intolerant adults [14]. In a systematic review, it was reported that aerobic exercise had a beneficial effect on cognitive function, especially on hearing, eyesight, and cognitive speed. They suggested that these beneficial effects could be resulted from improved of the cardiovascular system caused by aerobic physical activity [39].

The results of this study indicated that there was a significant positive correlation between exercise duration and MMSE scores. The MMSE scores are increased with increasing the frequency and duration of exercise. A prospective study of a representative rural community sample aged 65 and over, indicated that high intensity exercising for more than 5 days per week was negatively associated with cognitive decline [22]. These results and the results of the present study showed that exercising may have a similar effect on cognition for diabetic patients as well as general population. A decrease in duration or intensity of physical activity results in cognitive decline [40].

In this study it was estimated that the likelihood of cognitive decline was 2.28 times higher with increasing BMI. Not many studies have assessed the effect of exercising on cognitive status in overweight/obese type 2 diabetic patients. Some studies showed that increased risk of cognitive impairment in obese old diabetics [6] which was in line with our results. In addition, a number of epidemiological studies suggest that modification of vascular risk factors, such as hypertension [18], metabolic control, hypercholesterolemia [19] along with lowering hemoglobin A1C levels [17] in type 2 diabetic patients may be helpful in slowing down the progression of cognitive impairment. A longitudinal study on 253 type 2 diabetic patients with no dementia and 440 healthy individuals, showed that total fat mass and central adiposity may increased the risk of cognitive decline in older patients with type 2 diabetes [6].

In this study there was no significant correlation between MMSE scores and BMI in active group. In the controls however a significant correlation was reported. This may be explained by the positive effects of exercise on cognition. Among cognition impairment risk factors, obesity is a modifiable one. With weight control, patients with diabetes may delay or alter cognitive decline.

This study revealed that the likelihood of cognitive decline is 8.01 times higher with increasing age. Recent studies on older adults with normal cognition showed that low-intensity level mind-body and endurance exercise training may reduce the progression of age-related cognitive decline assessed by MMSE, respectively [41,42]. These results are in accordance with the present study in which the active group had higher MMSE scores. In addition women with senile dementia who regularly exercised 30–60 minutes per day, 2–3 times per week for 12 months, had higher MMSE scores compared to controls [20]. Regular exercising may improves senile dementia through not only lowering the chance of developing cognitive decline in old ages, but also may promote cognitive ability even in dementia patients.

In this study the majority of the participants 44(55%) did regular exercise during the past 6–9 years. A population- based case- control study showed that getting engaged in any moderate-intensity exercise during midlife or older ages reduces the risk of developing mild cognitive impairment [21]. Another population-based cohort revealed that midlife physical activity may reduce or delay the risk of experiencing dementia later on in life [43]. The exercise interventions should be explored as a potential strategy for delaying dementia onset [44]. The risk of cognitive impairment may be decreased after any exercise performed from adulthood up to elderly.

In this study it was estimated that the chance of developing cognitive decline was 1.25 times higher with increasing duration of diabetes. Diabetes and its duration were accompanied with cognitive decline in both men and women [45]. The study of the cognitive status of 16596 women aged between 70 and 81 years old indicated that the risk of experiencing cognitive decline in women with type 2 diabetes was increased with insulin use and the duration of diabetes [46].

It was also estimated that the chance of experiencing foot self care practice decreased 1.99 times with cognitive decline. The better means of foot self care practice in the active group may be due to their better cognitive function. As discussed above being diabetic and overweight may due to cognitive dysfunction and exercise may improve cognition. Results of a case control study for determining the effects of cognitive impairment on self-care behavior on diabetics patients and age and sex matched healthy adults showed higher rate of cognitive dysfunction in elderly subjects with predominantly type 2 diabetes, adding that the poor mental status in these patients was also associated with poor ability in diabetes self-care and greater dependency [47].

In another cross-sectional study on 50 adults with type 2 diabetes and 50 control subjects without diabetes revealed that middle-aged adults with type 2 diabetes manifest psychomotor slowing that is associated with poor metabolic control but, learning memory and problem solving skills were unaffected [48]. However the mean age of the participants in our study was higher and the results of our and other studies showed that cognitive status decrease with increasing age.

There was a significant difference between the mean score of foot self care practice in the two groups as the participants in the active group did better foot care practice. A review article on 32 studies assessing the effect of type 2 diabetes on cognitive function, reported that diabetic patients performed more poorly in neuropsychological tests [49].

The results of a study by Bell et al. showed that 23% of the participants did not check their feet [50]. In our study however, 16.2% of the active group and 11.2% of the controls checked their feet in 0–3 days in a week. The higher rate in the active group could be explained by their higher attention to foot care practice. Different instruments was used in different studies so, the findings cannot comparable.

The results of this study showed that in the both group the foot self care practice decreased in participants whose BMI was between 29–29.9, may indicating that increasing weight is also associated with reduced cognitive status and foot self care.

The major limitation of the present study is its cross-sectional nature and the fact that the information on the patients’ exercise and foot care practice was gathered through self reporting. Longitudinal studies are required to clarify the relation between exercise and cognitive function.

## Conclusion

The results revealed that regular exercising helps improve the cognitive status in overweight patients with type 2 diabetes, adding that the adherence to foot self care practice decreased with cognitive decline. Regular exercising may delay cognitive decline due to presence two concomitant risk factors of cognitive impairment; type 2 diabetes and overweight that improve foot self care. The results can help health care provider when counseling people with type 2 diabetes about self-care plan and exercise.

## Competing interests

The authors declare that they have no competing interests.

## Authors’ contributions

FM was involved in Study conception/design, acquisition of data, data analysis, interpretation of data and drafting the article. MH was involved in Study conception/design, drafting the article, Critical revisions for important intellectual and final approval of the version to be published. Dr MK was involved in Study conception/design, Supervision, Data collection, Critical revisions for important intellectual and final approval of the version to be published. All authors read and approved the final manuscript.
